# Interaction Effects in a 1D Flat Band at a Topological Crystalline Step Edge

**DOI:** 10.1021/acs.nanolett.2c03794

**Published:** 2023-03-27

**Authors:** Glenn Wagner, Souvik Das, Johannes Jung, Artem Odobesko, Felix Küster, Florian Keller, Jedrzej Korczak, Andrzej Szczerbakow, Tomasz Story, Stuart S. P. Parkin, Ronny Thomale, Titus Neupert, Matthias Bode, Paolo Sessi

**Affiliations:** † Department of Physics, University of Zurich, Winterthurerstrasse 190, 8057 Zurich, Switzerland; ‡ Max Planck Institute of Microstructure Physics, Halle 06120, Germany; § Physikalisches Institut, Experimentelle Physik II, Universität Würzburg, Am Hubland, 97074 Würzburg, Germany; ∥ Institute of Physics, Polish Academy of Sciences, Aleja Lotników 32/46, 02-668 Warsaw, Poland; ⊥ International Research Centre MagTop, Institute of Physics, Polish Academy of Sciences, Aleja Lotników 32/46, 02-668 Warsaw, Poland; # Institut für Theoretische Physik und Astrophysik Universität Würzburg, 97074 Würzburg, Germany

**Keywords:** topological crystalline insulators, Hartree−Fock, topological edge states, strong correlations in flat bands

## Abstract

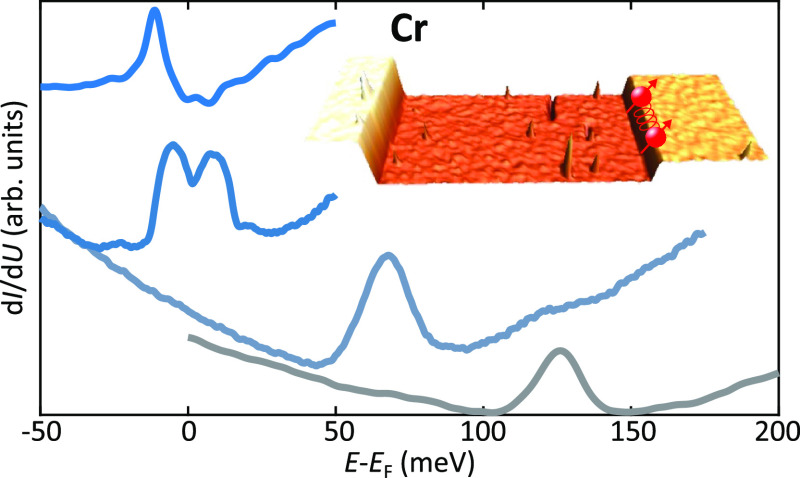

Step edges of topological crystalline insulators can be viewed as predecessors of higher-order topology, as they embody one-dimensional edge channels embedded in an effective three-dimensional electronic vacuum emanating from the topological crystalline insulator. Using scanning tunneling microscopy and spectroscopy, we investigate the behavior of such edge channels in Pb_1–*x*_Sn_*x*_Se under doping. Once the energy position of the step edge is brought close to the Fermi level, we observe the opening of a correlation gap. The experimental results are rationalized in terms of interaction effects which are enhanced since the electronic density is collapsed to a one-dimensional channel. This constitutes a unique system to study how topology and many-body electronic effects intertwine, which we model theoretically through a Hartree–Fock analysis.

The hallmark feature of three-dimensional topological insulators (TIs)^1,2^ are their protected gapless surface states with the dispersion of an *odd* number of massless Dirac Fermions. These surface states have a property called *chirality*, which makes them anomalous: It is not possible to obtain these two-dimensional surface states without incorporating the three-dimensional bulk. Mathematically, this is encoded in the Fermion doubling theorem^3−5^ which says that it is not possible to obtain Fermions of a single chirality in a purely two-dimensional system with time-reversal.

Topological crystalline insulators (TCI) are TIs that are protected by crystalline symmetries.^6,7^ In contrast to TIs, the surface of this TCI can host *multiple* Dirac cones, which are all of the *same* chirality (see Figure 1a), and exhibit the rotation anomaly: A purely two-dimensional model would have an equal number of Dirac cones with positive and negative chirality (see Figure 1b).^8^ In this work we investigate the one-dimensional edge states arising at odd-atomic step edges on the surface of the TCI Pb_1–*x*_Sn_*x*_Se (Figure 1c). The detection of these spin-polarized midgap states at step edges on the surface of Pb_1–*x*_Sn_*x*_Se was described in previous work including some of the present authors,^9^ which was confirmed in ref (10) and further theoretically detailed in ref (11). In this contribution, we report scanning tunneling microscopy (STM) and spectroscopy (STS) measurements of these edge states using surface doping to controllably tune their energy position with respect to the Fermi level. This experimental approach is used to systematically scrutinize the emergence of correlation effects under the effect of distinct dopants.

**Figure 1:**
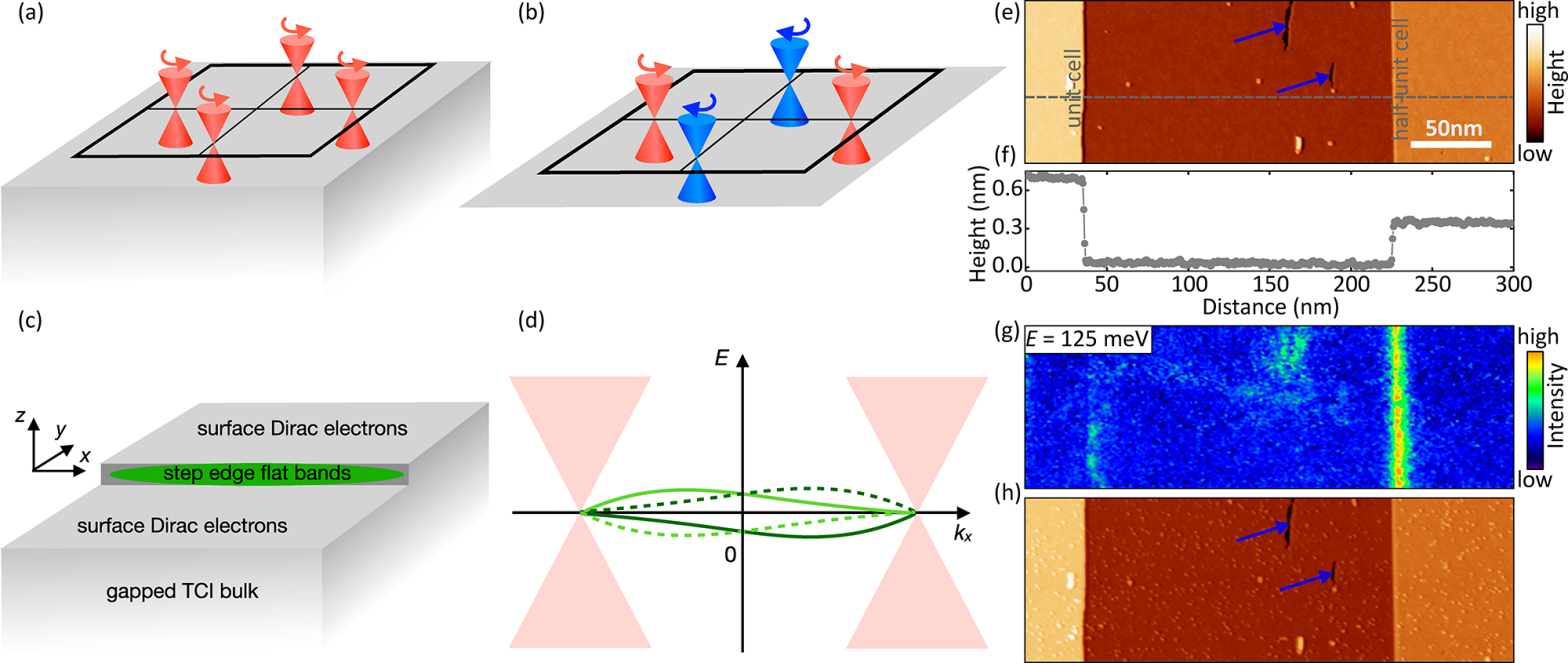
Emergence of 1D flat bands in TCI. (a) The TCI Pb_1–*x*_Sn_*x*_Se has four Dirac cones of the same chirality in the BZ. (b) For a purely 2D system there would be an equal number of Dirac cones with positive and negative chirality. (c) 1D flat bands emerge at a step edge on the TCI surface. (d) Band structure eq 3 of the four edge mode states along with the surface Dirac cones. (e) STM topographic image acquired at the (001) surface of pristine Pb_0.7_Sn_0.3_Se. The dashed gray line corresponds to the line profile reported in panel f. Two different steps are visible, corresponding to unit and half-unit cell heights. (g) d*I*/d*U* map acquired at the Dirac point (*E*_D_ = +125 meV). The signal, proportional to the sample local density of states (LDOS), shows a strong enhancement localized around the half unit cell step. Scanning parameters: *V* = 125 mV, *I* = 250 pA, *V*_rms_ = 10 meV. (h) STM topographic image of the very same sample region reported in panel e after Cr adatoms have been deposited onto the surface.

In typical 3D TIs the Coulomb interaction is not strong enough to lead to spontaneous symmetry breaking in the two-dimensional surface states.^12^ For Pb_1–*x*_Sn_*x*_Se with its large dielectric constant which effectively screens electron–electron interactions, correlation effects are generally disregarded.^13^ However, the 1D flat bands, which reside at step edges, are characterized by an enhanced density of states which can lead to correlated states (Figure 1d). For example, in an attempt to provide a possible explanation for the zero-bias conductance peak observed in point contact spectroscopy experiments,^14^ it has been suggested that 1D flat bands might be susceptible to correlation-driven instabilities resulting in the formation of magnetic domains.^15^ Similar flat boundary states are known to arise in a variety of systems, such as graphene,^16^ topological semimetals,^17^ and d-wave superconductors,^18^ which in some cases exhibit spontaneous symmetry breaking. In the present case, the edge modes have a flat dispersion and are therefore susceptible to flat-band Stoner ferromagnetism—a one-dimensional analogue of quantum Hall ferromagnetism in the zeroth Landau level (LL) of graphene^19,20^ or in twisted bilayer graphene.^21,22^

Spontaneous symmetry breaking is associated with the opening of correlation gaps. Our spectroscopic measurements show the following behavior, when the energy of the 1D flat band is tuned to the Fermi level, the single peak in the density of states (DOS) from the edge mode splits into either two or four peaks. A theoretical assessment within *k*·*p* theory explains the different experimentally observed peak multiplicities by a variation of the ratio *V*/*W*, where *V* is the interaction energy and *W* is the bandwidth, resulting in up to four states that spontaneously break time-reversal symmetry.

Pb_1–*x*_Sn_*x*_Se crystallizes in a rock salt structure for *x* ≤ 0.4. Previous studies showed how this substitution alloy can host two topological distinct phases.^23^ Starting from PbSe, a trivial narrow band gap semiconductor, the system undergoes a topological phase transition by progressively increasing the Sn concentration. At low temperature, the topological crystalline phase is observed for *x* ≥ 0.2.^24^ In the present study, we focus on Pb_0.7_Sn_0.3_Se single crystals grown by the self-selecting vapor growth method.^9,24^ Our crystals are thus safely inside the topological crystalline regime of the Pb_1–*x*_Sn_*x*_Se phase diagram. Single crystals have been cleaved at room temperature in ultrahigh-vacuum conditions (*p* < 5 × 10^–10^ mbar). Experiments have been performed in two distinct STM setups, operated at *T* = 2 K and *T* = 4.5 K. All measurements have been acquired using electrochemically etched tungsten tips. Differential conductance d*I*/d*U* data have been measured by a lock-in technique by applying a bias voltage modulation *V*_rms_ to the tip.

Figure 1e shows an STM topographic image acquired in constant-current mode on a freshly cleaved Pb_0.7_Sn_0.3_Se crystal. The exposed surface corresponds to the (001) orientation which is commonly obtained when cleaving a bulk crystal.^24−28^ At this surface, angle-resolved photoemission studies revealed the presence of four Dirac cones protected by mirror symmetry located close to the *X̅* and *Y̅* points of the Brillouin zone.^24−26,29^ The topographic image shows large terraces separated by step edges which, as highlighted by the line profile reported in Figure 1f, are characterized by different heights. These two steps are representative of two distinct classes, namely, (i) steps whose height is equal to an integer multiple of the lattice constant *n*·*a*, and (ii) steps whose height is a half-integer multiple of the lattice constant (1/2 + *n*)*a* with *n* being the integer and *a* the lattice constant (*a* ≈ 6 Å). As described in ref (9), while the translation symmetry of the surface lattice is preserved for integer multiple steps, half-integer multiple steps introduce a 1D structural π-shift which dramatically influences the surface electronic properties. This is illustrated in Figure 1g, which reports a d*I*/d*U* map acquired at the Dirac point located at *E*_D_ ≈ + 125 meV (see Supporting Information Figure 1 for a description of the energy level alignment). The d*I*/d*U* signal, which is proportional to the sample LDOS, shows a strong enhancement at the half-integer step. As discussed in ref (9), this corresponds to the spectroscopic signature of a 1D flat band localized around the 1D structural π-shift.

The present system thus represents an ideal platform to scrutinize the emergence of interaction effects in 1D flat bands, which are expected to manifest once the flat bands are energetically localized close to the Fermi level. The key idea is that, as the kinetic energy is quenched, electron correlations can become the dominant energy scale. To experimentally realize such a scenario, the 1D flat band has to be tuned to the Fermi level. To achieve this goal, we used a surface doping approach. Starting from pristine p-doped crystals, we progressively dose higher amounts of distinct 3d adatoms onto the crystal surface held at cryogenic temperature, a procedure known to create a downward band bending, i.e. a rigid shift toward negative energies.^30^ This procedure is illustrated in Figure 1h, which shows a topographic image of the very same sample region reported in Figure 1e after Cr adatoms have been deposited on the surface. Close inspection of the image reveals small protrusions which correspond to Cr adatoms. Representative images acquired after dosing Mn, Fe, and Cu adatoms are reported in Supporting Information Figure 2.

Figure 2a–d summarizes the spectroscopic results as a function of the doping level, with each panel corresponding to a distinct dopant, namely Cr, Mn, Fe, and Cu. Starting from pristine samples (gray lines), a rigid shift toward negative energy is observed upon deposition onto the surface, irrespective of which specific 3d element is used. The shift successively increases with each deposition step. Once the 1D flat band is close to the Fermi level, the single peak is found to split into a double-peak structure, as highlighted by the insets in Figure 2a–d. By further increasing the concentration of surface dopants, the Dirac point is shifted below the Fermi level which results in the recovery of a single peak structure characteristic of the 1D flat band.

**Figure 2:**
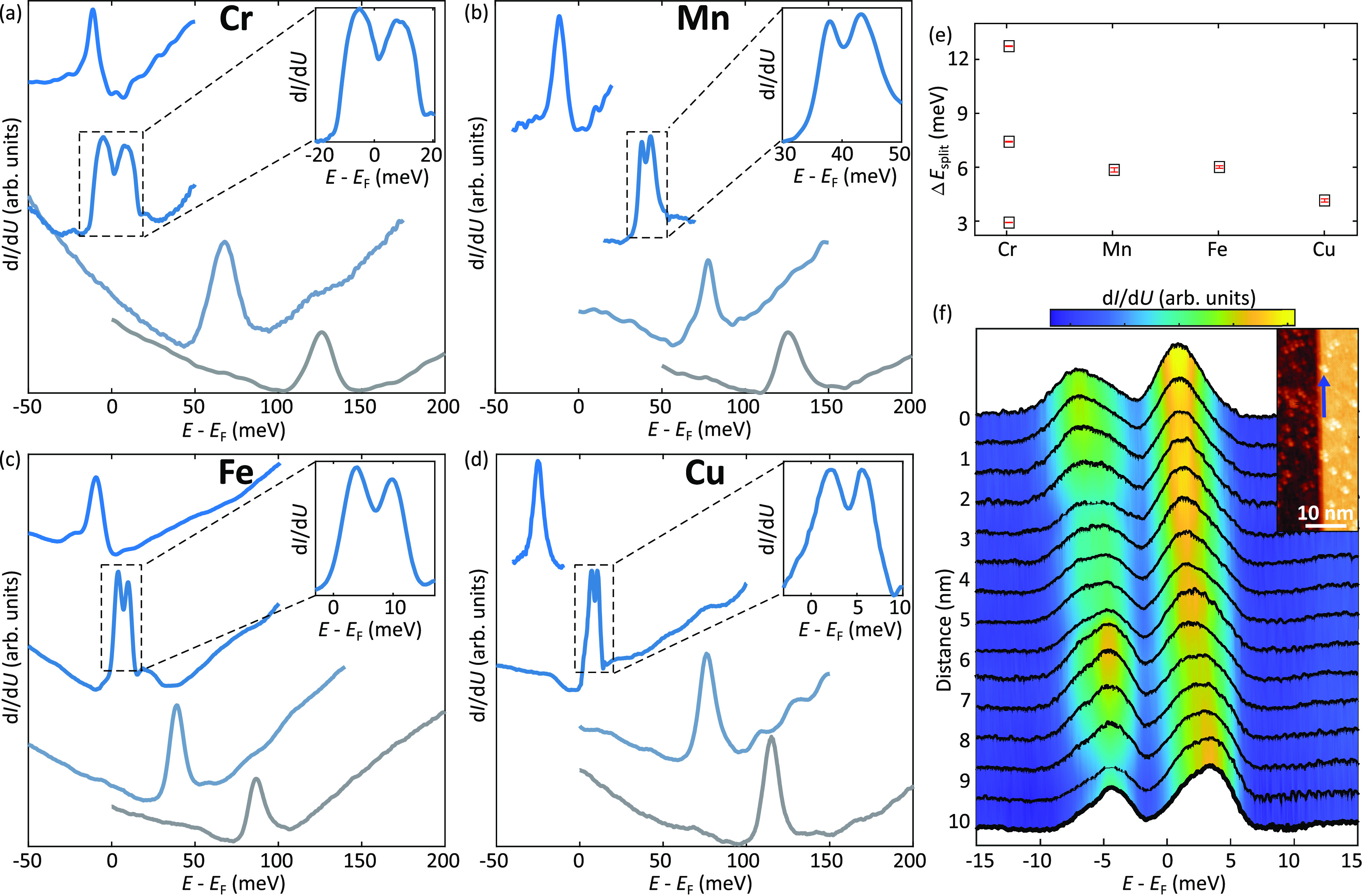
Doping dependence of the 1D flat band. (a–d) Scanning tunneling spectroscopy of the 1D flat band emerging at half unit cell steps as a function of the doping level. Each panel reports the energy evolution of the 1D flat band at different doping concentrations for distinct dopants (Cr, Mn, Fe, and Cu). Measurements on Cr- and Mn-doped samples have been performed at *T* = 2 K. Measurements on Fe- and Cu-doped samples have been performed in a different setup operated at *T* = 4.5 K. For all elements, the deposition onto the Pb_0.7_Sn_0.3_Se surface provides a n-doping effect. Starting from p-doped crystals, this procedure allows to progressively shift the energy of the 1D flat band toward the Fermi level. A splitting of the single peak in LDOS into a double-peak structure is visible once the 1D flat band is energetically close to the Fermi level, as highlighted in the insets. By continuously doping the surface, the splitting disappears once the 1D flat band is shifted below the Fermi level. (e) Magnitude of the splitting observed in panels a–d. (f) Line spectroscopy acquired along a step edge once the energy of the 1D flat band is brought close to the Fermi level (Cr adatoms as dopants). The inset shows a STM topographic image of the step edge along which the line spectroscopy (marked by the blue arrow) has been acquired. The spectra provide evidence for the existence of spatial fluctuations of the double-peak structure, an effect attributed to intrinsic sample inhomogeneities and the disorder created by the random distribution of dopants.

In all cases, the size of the splitting amounts to a few meV, as summarized in Figure 2e. The different data points reported for Cr correspond to distinct experimental runs, revealing a distribution in the size of the splitting which is not linked to the specific element but which is attributed to sample inhomogeneities (both intrinsic as well as induced by the random distribution of dopants), which can affect the flatness of the 1D bands (see theory section). This is demonstrated by the additional spectroscopic data reported in Supporting Information Figures 3 and 4.

To test the robustness of this observation against potential artifacts, we performed numerous control experiments. For example, in order to exclude an uncontrolled influence of a spatial inhomogeneity of the TCI surface, the very same sample region was mapped before and after deposition, as illustrated in Figure 1. Moreover, we verified that integer step edges under the same doping conditions, i.e. once the Dirac point is tuned to the Fermi, do not show any significant change with respect to the spectral shape observed in the pristine case, see Supporting Information Figure 5. This ensures that the observed behavior is indeed linked to the evolution of the electronic properties of the 1D flat band hosted at half-integer steps as a function of doping level.

Note that, although this surface doping approach allows us to controllably shift the Dirac point toward the Fermi level, the random distribution of dopants inevitably increases the surface disorder after each deposition step. This results in spatial fluctuations of the Dirac point illustrated in Figure 2f, which reports spatially resolved scanning tunneling spectroscopy acquired at distinct positions along a structural π-shift (see blue arrow in the inset of Figure 2f). Although these data provide evidence of the existence of different broadening as well as fluctuations in the peak intensity, the peak splitting remains clearly visible along the entire profile.

A splitting of the 1D flat band into a double-peak structure is predominantly found in our samples once the Dirac point is close to the Fermi level. However, our measurements frequently reveal the existence of a spectroscopically more rich scenario where the LDOS associated with the 1D flat band splits into a multipeak structure. This is illustrated in Figure 3, which reports three representative spectra acquired on different samples. As for the double-peak case discussed in Figure 2f, the spectra reveal a clear suppression of the LDOS near the Fermi. However, each peak has been further split into two subpeaks, resulting in a four-peak structure. We note that, while a dip at the Fermi is always clearly detected, the splitting of each peak into a doublet is more subtle and its observation can be easily hampered by broadening, both intrinsic (related to the bandwidth of 1D flat band) as well as disorder-induced (related to random distribution of dopants). As discussed in the theory section, these observations are in agreement with our theoretical analysis, being a direct fingerprint of two distinct energy scales.

**Figure 3:**
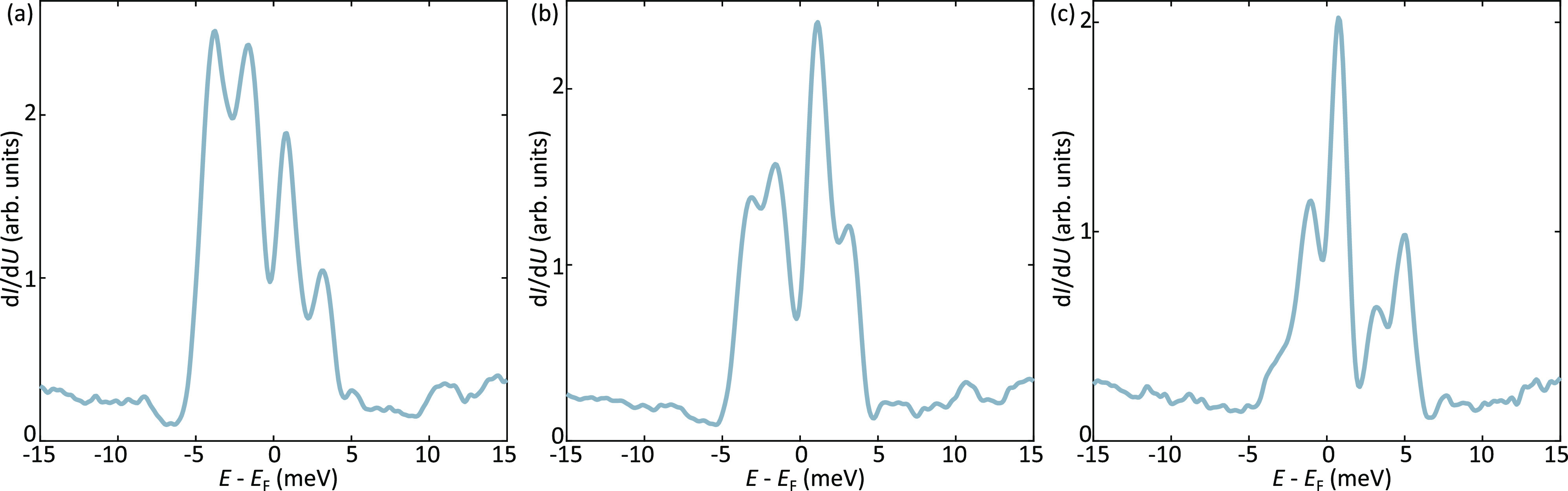
Spectroscopic signatures of interaction effects. (a–c) Scanning tunneling spectroscopy data acquired at odd-atomic step edges which exhibit a structural π-shift. The measurements have been acquired on different samples. Cr adatoms have been used as dopants. Similar to the double-peak structure discussed in Figure 2, a clear suppression of the LDOS is visible at the Fermi level. Additionally, each peak splits into a doublet, resulting in a four-peak structure.

The *k*·*p* theory for Pb_1–*x*_Sn_*x*_Se has been worked out in refs (31 and 32), and the corresponding Landau level spectrum was discussed in ref (33). Here, as a model we propose a more simple Hamiltonian consisting of four Dirac points in the BZ at (±κ, ±κ):
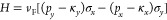
1where *p*_*i*_ = −*i∂*_*i*_ and σ_*i*_ are the Pauli matrices associated with spin. We label the valleys by two pseudospin degrees of freedom τ_*i*_, η_*i*_. The step edge manifests as an exchange of the valleys between *y* > 0 and *y* < 0, such that *y* = 0 is the location of the step edge (see Figure 4a): (κ_*x*_, κ_*y*_) = κ(τ_*z*_ sign(*y*), η_*z*_). Estimates for the Fermi velocity *v*_F_ can be found in refs (34 and 35).

**Figure 4:**
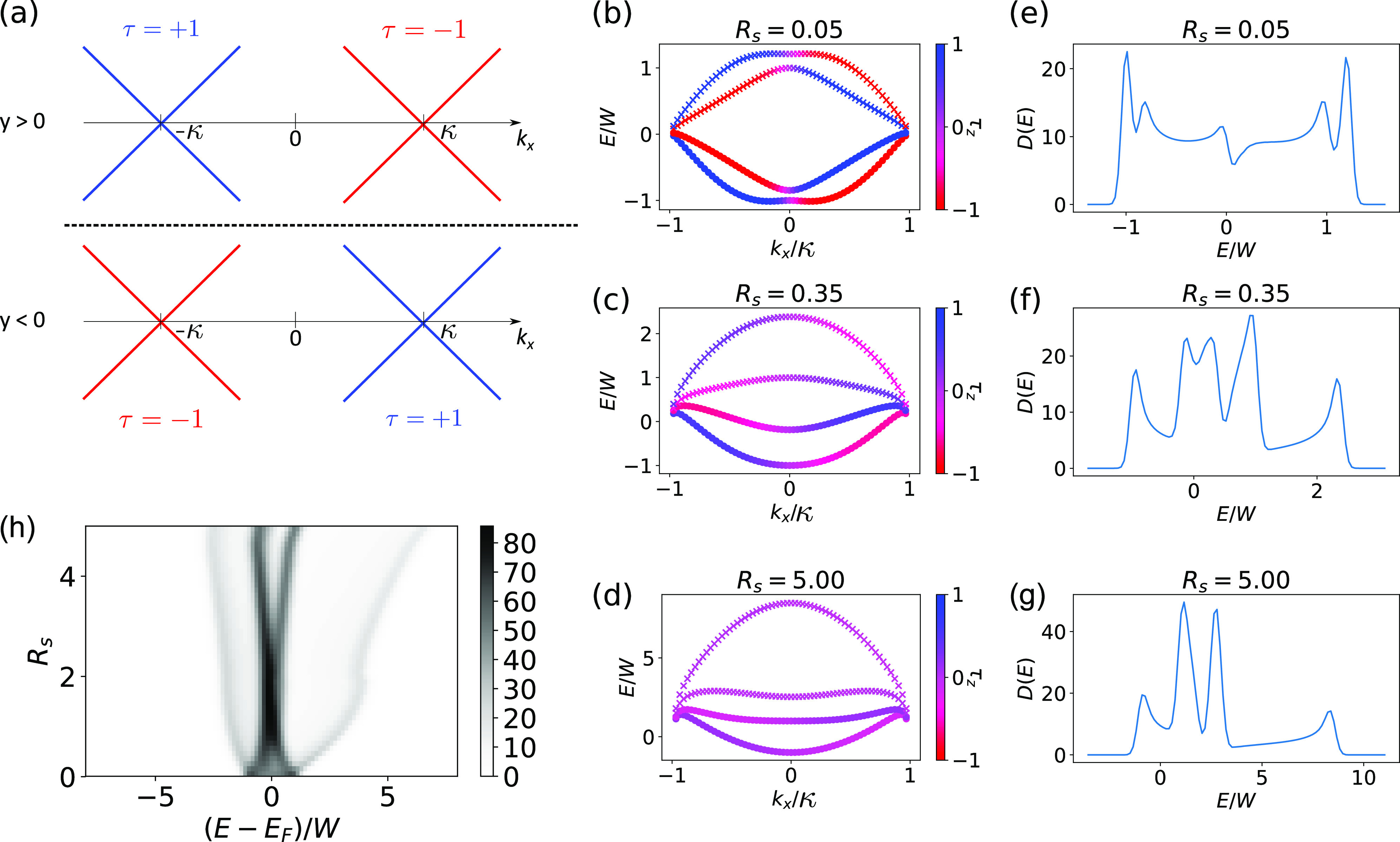
Model and Hartree–Fock results. (a) Schematic of the toy model in eq 1 we consider for the step edge. We shift the two Dirac cones of the two valleys by an amount κ along the *x*-axis. We take this shift to be opposite for *y* > 0 and *y* < 0; this way we obtain an edge mode at *y* = 0. The HF band structures (b–d) along with the corresponding DOS (e–g) are shown for three values of *R*_*s*_ and κ̅ = 1. The color bar in the band structure shows ⟨τ_*z*_⟩. As *R*_*s*_ increases, we obtain a two- and four-peak structure. The conduction and valence band are split by the energy scale *W*. Hybridization between orbitals leads to interaction-induced gaps of order *V* opening up. The circles indicate filled states while the crosses indicate empty states. We pick a resolution of *N*_*y*_ = 41 for *k*_*y*_. (h) Hartree–Fock results showing the continuous evolution of the DOS as a function of the interaction parameter *R*_*s*_ = *V*/*W*. As *R*_*s*_ increases from zero, the four peaks are initially so close that they will not be resolved due to the thermal smearing in the experiment. However, for larger *R*_*s*_, the four peaks can clearly be distinguished. In experiments, either four-peak or two-peak structures are observed at the Fermi energy. There are inhomogeneities within the sample, and due to this disorder, the bandwidth *W* can vary from sample to sample and also within a sample from one position to another. This explains the observation of either two or four peaks seen in the experiments at the Fermi energy, since the value of *R*_*s*_ may be different in the two cases.

We label the eigenstates by their σ_*z*_, τ_*z*_, η_*z*_ eigenvalues σ, τ, η. There are four zero modes in the range −κ < *k*_*x*_ < κ which are localized around *y* = 0 with opposite spins in the two valleys (i.e., the eigenvalue of σ_*z*_ is the same as the eigenvalue of τ_*z*_^36^):
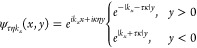
2

We study the symmetry-breaking patterns in the edge states due to electron–electron interactions. This problem is reminiscent of the long-standing problem of magnetism in graphene edges. The zigzag edge of graphene hosts an exact zero-energy mode^37,38^ (a finite dispersion for the edge modes can be generated by next-nearest neighbor hopping), and interactions lead to a ferromagnetic state, as shown in Hartree–Fock,^37^ exact diagonalization,^39^ perturbative approaches,^40^ and bosonization.^41^ Furthermore, in graphene nanoribbons, the two edges can be coupled by interactions, leading to antiferromagnetic interedge coupling.^37,42,43^ By a similar mechanism, the Majorana flat bands in d-wave superconductors order magnetically.^44^ We choose to study the step-edge problem in a similar vein and rely on the Hartree–Fock approximation, since for zigzag edges more sophisticated techniques yield similar results. There are two important differences between the zigzag edges of graphene and the step-edge modes studied here. First, we have twice the number of flat bands, namely four instead of two. Second, unlike in graphene the edge modes in the TCI are not spin-degenerate since Pb_1–*x*_Sn_*x*_Se exhibits a significant spin–orbit coupling.

In the phenomenological model introduced above, we obtain fully flat bands for the edge states. However, a microscopic three-dimensional model finds edge states with a finite dispersion;^9^ hence, we add this dispersion by hand. The bands calculated in ref (9) have two van Hove singularities (VHSs). One of the VHSs arises where the flat band merges with the Dirac cone, at which point the states also get more extended perpendicular to the edge. Therefore, we expect only the other VHS to show up as a peak in the edge density of states (DOS) measured by the STS. This motivates the following model for the dispersion (Figure 1d):
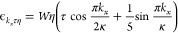
3with *W* being the bandwidth. The full second-quantized Hamiltonian is of the form *H* = *H*_kin_ + *H*_int_ where *H*_kin_ = ∑_α_ϵ_α_*c*_α_^†^*c*_α_, and the interaction term will be
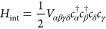
4where we use the short-hand label α = (*k*_*x*_, τ, η). The matrix elements *V*_*αβγδ*_ are obtained by projecting the Coulomb interaction onto the flat bands. Since our model is a purely two-dimensional model of the surface, we use the two-dimensional Coulomb interaction . Screening from the three-dimensional bulk may result in a renormalized dielectric constant. We perform a mean-field decoupling of the Hamiltonian and solve the Hartree–Fock equations self-consistently (see Supporting Information for details).

There are two energy scales in the problem. The kinetic energy scale is the bandwidth *W*, while the interaction energy scale is . The model thus has two dimensionless parameters, κ̅ = *κa* (*a* is the lattice spacing in the *y*-direction) and *R*_*s*_ = *V*/*W*. The qualitative results are largely independent of κ̅; for the band structure of the TCI in question in this work, we have κ̅ = 0.5.^9^ Rather, we focus on the dependence on *R*_*s*_. Let us consider this model at half filling. The HF results are shown in Figure 4. In the limit *R*_*s*_ ≪ 1, we completely fill the valence band subspace (η = −1), and the interaction leads to a slight hybridization between the opposite spin bands at the band crossing (Figure 4b). This state spontaneously breaks time-reversal symmetry and leads to two peaks in the DOS (Figure 4e), with a splitting given by *W*. In the limit *R*_*s*_ ∼ 1, the splitting between the conduction and valence band (∼*W*) remains, and the valence band subspace is completely filled. Due to the interaction, however, the opposite spin bands in the valence and conduction band subspaces are fully hybridized forming bonding and antibonding orbitals, which are split by an amount *V* (Figure 4c), thus leading to four peaks in the DOS (Figure 4f). For *R*_*s*_ ≫ 1 the kinetic term is negligible, and there is mixing between all four bands, again forming bonding and antibonding orbitals (Figure 4d). Since we can form bonding and antibonding orbitals in both the spin and the conduction/valence band degrees of freedom, this leads to a four-peak DOS (Figure 4g), where the splitting is set by *V*. We show the continuous evolution of the DOS as a function of *R*_*s*_ in Figure 4h, demonstrating that the interaction continuously splits the two peaks into a four-peak structure.

We used a combination of high-resolution STM and theoretical calculations to investigate the edge modes arising at a step edge on the surface of the topological insulator Pb_1–*x*_Sn_*x*_Se. We developed a continuum model description of these edge states and performed a Hartree–Fock calculation to investigate the effect of interactions. The edge modes have a flat dispersion, thus leading to ferromagnetic states, which may open up additional correlation gaps, as seen in the STM measurements on the system when doped to the Fermi level. In future work, it would be interesting to perform spin-resolved STM measurements on the edge modes to confirm that edge modes follow the symmetry-breaking patterns predicted by the HF calculation.

The step-edge flat bands studied here have similarities to the edge states arising at the zigzag edge of graphene. In graphene nanoribbons, the edges can be close enough such that they are coupled via interactions. In that case it is known that while the intraedge coupling is ferromagnetic, the interedge coupling is antiferromagnetic. It is therefore natural to wonder what would happen with two nearby step edges in the TCI and how the edge modes are then coupled. Previous work has shown that two nearby step-edge modes can couple to form bonding and antibonding orbitals.^28^ It remains an open question what happens to the magnetism in that case.

## Supporting Information Available

Additional experimental data and details on the Hartree–Fock calculation (PDF)
