# Supplementary material for: Interaction Effects in a 1D Flat Band at a Topological Crystalline Step Edge
Source: Nano Lett. 2023 Mar 27;23(7):2476–82. doi: 10.1021/acs.nanolett.2c03794 (PMC10103314; doi:10.1021/acs.nanolett.2c03794)
Supplement: Supplementary file 1 — nl2c03794_si_001.pdf [file nl2c03794_si_001.pdf]

— Supplementary Material —
Interaction effects in a 1D flat band at a topological crystalline step edge

Glenn Wagner,[1,*] Souvik Das,[2,*] Johannes Jung,[3] Artem Odobesko,[3,†] Felix Küster,[2] Florian Keller,[3] Jedrzej Korczak,[4,5] Andrzej Szczerbakow,[4] Tomasz Story,[4,5] Stuart S.P. Parkin,[2] Ronny Thomale,[6] Titus Neupert,[1] Matthias Bode,[3] and Paolo Sessi[2]

[1]*Department of Physics, University of Zurich, Winterthurerstrasse 190, 8057 Zurich, Switzerland*
[2]*Max Planck Institute of Microstructure Physics, Halle 06120, Germany*
[3]*Physikalisches Institut, Experimentelle Physik II, Universität Würzburg, Am Hubland, 97074 Würzburg, Germany*
[4]*Institute of Physics, Polish Academy of Sciences, Aleja Lotników 32/46, 02-668 Warsaw, Poland*
[5]*International Research Centre MagTop, Institute of Physics, Polish Academy of Sciences, Aleja Lotników 32/46, 02-668 Warsaw, Poland*
[6]*Institut für Theoretische Physik und Astrophysik Universität Würzburg, 97074 Würzburg, Germany*

Appendix A: Supplementary figures

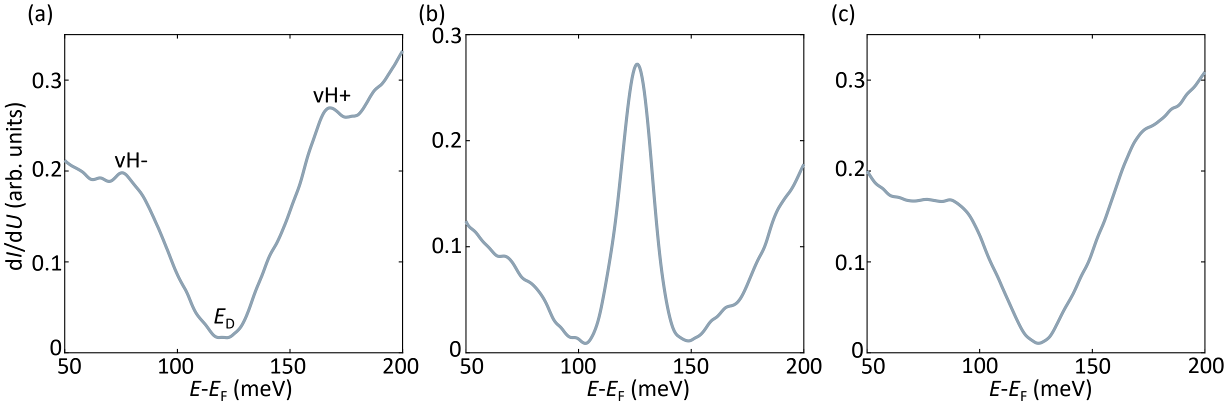

FIG. S1. Scanning tunneling spectroscopy data acquired by positioning the tip on (a) terrace, (b) step corresponding to a structural π shift, whose height corresponds to a half unit cell, and (c) step where translational invariance is preserved,corresponding to a unit cell height. vH- and vH+ identify the position of van Hove singularities energetically located at + 90 meV and + 175 meV,respectively. The minimum visible at $E_D \approx +$ 125 meV corresponds to the position of the Dirac point. At the energy corresponding to the Dirac point, a strong peak is visible in (b). As explained in Ref. 1, this peak is the spectroscopic signature of 1D flat bands localized around the step.

* These authors contributed equally to this work.

† Corresponding author, artem.odobesko@uni-wuerzburg.de

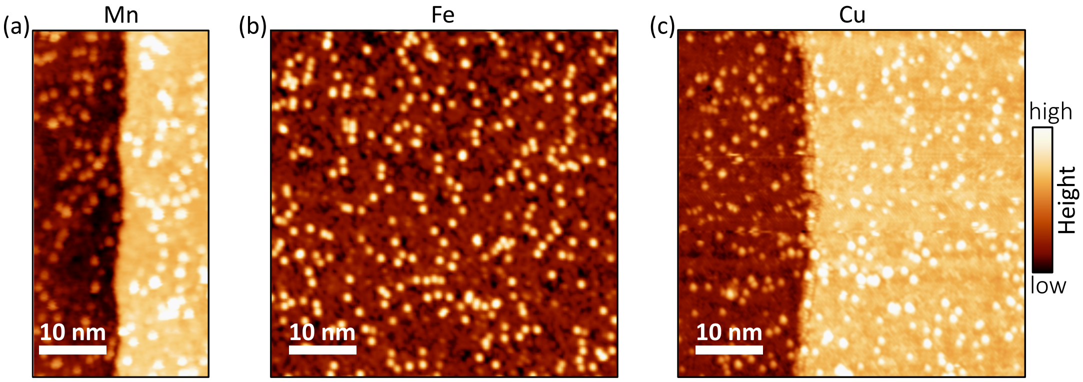

FIG. S2. STM topographic images of different $Pb_{1-x}Sn_xSe$ samples after Mn, Fe, and Cu adatoms have been deposited onto the surface. Close inspection of the images reveals small protrusions corresponding to the dopants.

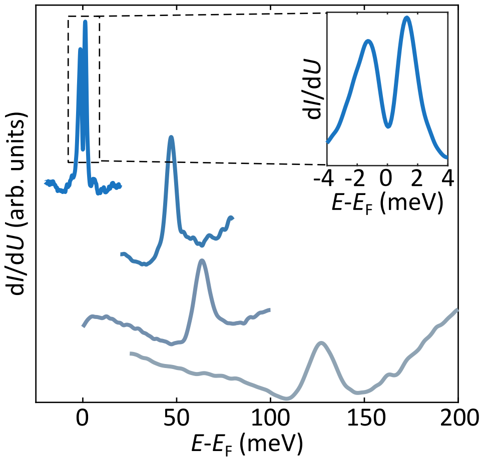

FIG. S3. Doping dependent energy shift. Scanning tunneling spectroscopy of the flat band emerging at half unit cell steps as a function of the doping level. Cr adatoms deposited on the $Pb_{0.7}Sn_{0.3}Se$ surface provide a n-doping effect. Starting from a p-doped crystal, this allows to progressively shift the energy of the 1D flat band towards the Fermi level. Although starting from a nominally equivalent sample, i.e. $Pb_{0.7}Sn_{0.3}Se$ with Dirac point located at $\sim$ 125 meV, the splitting detected once the 1D flat bands hits the Fermi, is lower (3 meV) with respect to the one reported in the inset of Fig. 2a (12.5 meV).

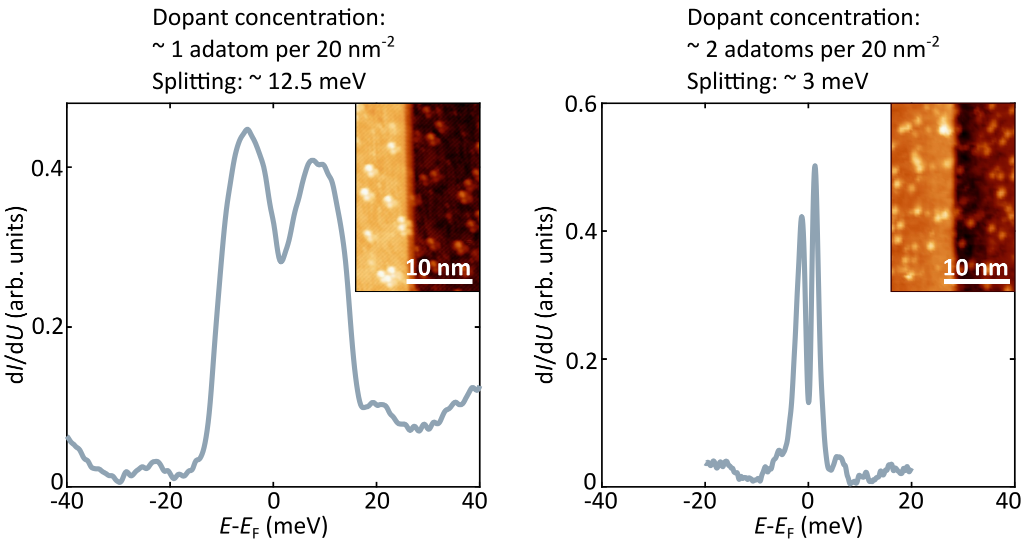

FIG. S4. Comparison of the size of the splitting for samples characterized by different surface dopants concentration. The insets report topographic images corresponding to the areas where the respective spectroscopic data have been acquired. In both cases, Cr adatoms have been used as dopants.

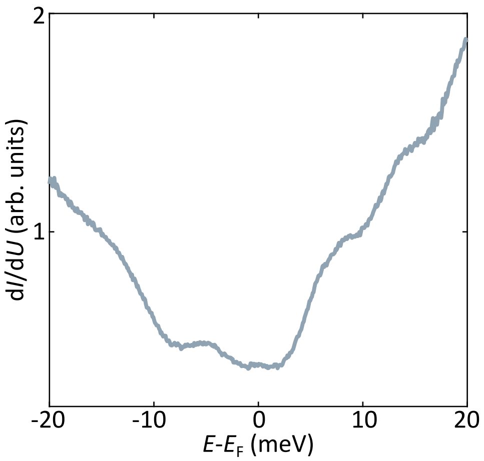

FIG. S5. Scanning tunneling spectroscopy acquired by positioning the tip on top of an integer step, i.e. a step with an height equals to the length of the unit cell. Surface dopants have been dosed to tune the Dirac point to the Fermi level. Contrary to spectra acquired on a structural π shift, the spectrum is characterized by a minimum at the Dirac point and it shows a similar line shape compared to the pristine case.

Appendix B: Hartree-Fock formalism

From the main text we know that the edge states are strips akin to Landau gauge orbitals in the quantum Hall effect, which have the form

$$\psi_{k_x\tau\eta}(x,y) \equiv \frac{1}{\sqrt{L_x}} e^{ik_x x} u_{k_x\tau\eta}(y) = \frac{1}{\sqrt{L_x}} e^{ik_x x + i\kappa\eta y} \begin{cases} e^{-|k_x - \tau\kappa| y}, & y > 0 \\ e^{|k_x + \tau\kappa| y}, & y < 0 \end{cases} \tag{S1}$$

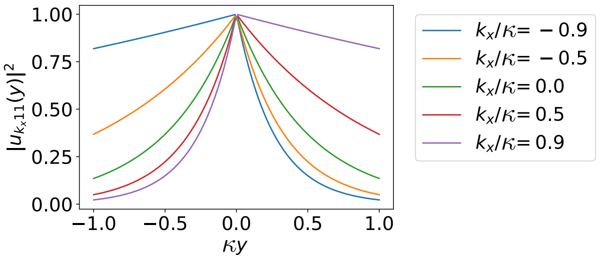

FIG. S6. The spatial profile of the wavefunctions in the direction perpendicular to the step edge for different momenta k_x. The mean position $\langle y \rangle$ of the wavefunction shifts from left to right as k_x increases. The maximum of the wavefunctions is always located at $y = 0$.

where $-\kappa < k_x < \kappa$. L_x is the length of the system in the x-direction. We have time-reversal and mirror symmetries

$$\mathcal{T} : \psi_{-k_x\bar{\tau}\bar{\eta}}(x,y) = \psi^*_{k_x\tau\eta}(x,y) \tag{S2}$$
$$\mathcal{M}_y : \psi_{k_x\tau\bar{\eta}}(x,y) = \psi_{k_x\tau\eta}(x,-y) \tag{S3}$$
$$\mathcal{M}_x : \psi_{k_x\bar{\tau}\eta}(x,y) = \psi_{-k_x\tau\eta}(-x,y) \tag{S4}$$
$$\mathcal{P} : \psi_{k_x\tau\bar{\eta}}(x,y) = \psi_{-k_x\tau\eta}(-x,-y) \tag{S5}$$

Note that time-reversal flips both spin τ and valley η and takes $k_x \to -k_x$. We want to project the Coulomb interaction down onto the Hilbert space of these edge states. The interaction term will be

$$H_{\text{int}} = \frac{1}{2} V_{\alpha\beta\gamma\delta} c^\dagger_\alpha c^\dagger_\beta c_\delta c_\gamma, \tag{S6}$$

where we use the short-hand label $\alpha = (k_x^\alpha, \tau^\alpha, \eta^\alpha)$ and the matrix elements are

$$V_{\alpha\beta\gamma\delta} = \langle \psi_\alpha \psi_\beta | \hat{V} | \psi_\delta \psi_\gamma \rangle \tag{S7}$$
$$= \int_{\mathbf{r},\mathbf{r}'} \psi^*_\alpha(\mathbf{r}) \psi^*_\beta(\mathbf{r}') V(\mathbf{r}-\mathbf{r}') \psi_\delta(\mathbf{r}) \psi_\gamma(\mathbf{r}') \tag{S8}$$
$$= \frac{1}{L_x^2} \sum_{q_x} \int_{q_y} \int_{\mathbf{r},\mathbf{r}'} e^{-ik_x^\alpha x - ik_x^\beta x' + ik_x^\delta x + ik_x^\gamma x' + iq_x(x-x') + iq_y(y-y')} V_q u^*_{k_x^\alpha \tau^\alpha \eta^\alpha}(y) u^*_{k_x^\beta \tau^\beta \eta^\beta}(y') u_{k_x^\delta \tau^\delta \eta^\delta}(y) u_{k_x^\gamma \tau^\gamma \eta^\gamma}(y'), \tag{S9}$$

where $V_q = \frac{2\pi}{L_x} \frac{e^2}{2\epsilon_0 q}$. We now define the (unnormalized) form factors

$$\tilde{\lambda}_{\alpha\delta}(k_x^\alpha, k_x^\delta, q_y) = \int_y u^*_{k_x^\alpha \tau^\alpha \eta^\alpha}(y) u_{k_x^\delta \tau^\delta \eta^\delta}(y) e^{iq_y y}. \tag{S10}$$

The form factors can be calculated analytically by using the expression (S1) and one finds

$$\tilde{\lambda}_{\alpha\delta}(k_x^\alpha, k_x^\delta, q_y) = \frac{i\kappa(\eta^\alpha - \eta^\delta) + iq_y + |k_x^\alpha - \tau^\alpha\kappa| + |k_x^\delta - \tau^\delta\kappa|}{(\kappa(\eta^\alpha - \eta^\delta) + q_y)^2 + (|k_x^\alpha - \tau^\alpha\kappa| + |k_x^\delta - \tau^\delta\kappa|)^2} + \frac{-i\kappa(\eta^\alpha - \eta^\delta) - iq_y + |k_x^\alpha + \tau^\alpha\kappa| + |k_x^\delta + \tau^\delta\kappa|}{(\kappa(\eta^\alpha - \eta^\delta) + q_y)^2 + (|k_x^\alpha + \tau^\alpha\kappa| + |k_x^\delta + \tau^\delta\kappa|)^2}. \tag{S11}$$

Note that the wavefunctions in (S1) are not normalized so the normalized form factors need to be calculated via

$$\lambda_{\alpha\delta}(k_x^\alpha, k_x^\delta, q_y) = \frac{\tilde{\lambda}_{\alpha\delta}(k_x^\alpha, k_x^\delta, q_y)}{\sqrt{\tilde{\lambda}_{\alpha\alpha}(k_x^\alpha, k_x^\alpha, 0)\tilde{\lambda}_{\delta\delta}(k_x^\delta, k_x^\delta, 0)}}. \tag{S12}$$

The form factors have the form

$$\lambda(k_x^\alpha, k_x^\delta, q_y) = (\Lambda^0(k_x^\alpha, k_x^\delta, q_y)\tau^0 + \Lambda^0(-k_x^\alpha, k_x^\delta, q_y)\tau^x)\eta_0 + (\Lambda^\times(k_x^\alpha, k_x^\delta, q_y)\tau^0 + \Lambda^\times(-k_x^\alpha, k_x^\delta, q_y)\tau^x)\eta_x. \tag{S13}$$

Then the matrix elements become

$$V_{\alpha\beta\gamma\delta} = \sum_{q_x}\int_{q_y} \delta_{q_x-k_x^\alpha+k_x^\delta,0}\delta_{-q_x-k_x^\beta+k_x^\gamma,0}\lambda_{\alpha\delta}(k_x^\alpha, k_x^\delta, q_y)\lambda^*_{\gamma\beta}(k_x^\gamma, k_x^\beta, q_y)V(q_x, q_y) \tag{S14}$$

$$= \delta_{k_x^\alpha+k_x^\beta=k_x^\gamma+k_x^\delta}\int_{q_y} \lambda_{\alpha\delta}(k_x^\alpha, k_x^\delta, q_y)\lambda^*_{\gamma\beta}(k_x^\gamma, k_x^\beta, q_y)V(k_x^\alpha - k_x^\delta, q_y), \tag{S15}$$

where the Kronecker delta in the last line is enforcing momentum conservation in the x-direction. Now we perform the mean-field decoupling of the Hamiltonian

$$H_{\text{int,HF}} = V_{\alpha\beta\gamma\delta}(c_\alpha^\dagger c_\delta\langle c_\beta^\dagger c_\gamma\rangle - c_\alpha^\dagger c_\gamma\langle c_\beta^\dagger c_\delta\rangle). \tag{S16}$$

A Slater determinant is described by the projector $P_{\alpha\beta} = \langle c_\alpha^\dagger c_\beta\rangle$. Assuming translational invariance along the x-direction, we have

$$P_{\alpha\beta}(k_x^\alpha, k_x^\beta) = \langle c_\alpha^\dagger(k_x^\alpha)c_\beta(k_x^\beta)\rangle = \delta_{k_x^\alpha,k_x^\beta}\langle c_\alpha^\dagger(k_x^\alpha)c_\beta(k_x^\alpha)\rangle \equiv \delta_{k_x^\alpha,k_x^\beta}P_{\alpha\beta}(k_x^\alpha). \tag{S17}$$

With this simplification the HF Hamiltonian becomes

$$H_{\text{int,HF}} = V_{\alpha\beta\gamma\delta}(c_\alpha^\dagger(k_x^\alpha)c_\delta(k_x^\alpha)P_{\beta\gamma}(k_x^\beta)\delta_{k_x^\beta,k_x^\gamma} - c_\alpha^\dagger(k_x^\alpha)c_\gamma(k_x^\alpha)P_{\beta\delta}(k_x^\beta)\delta_{k_x^\beta,k_x^\delta}) \tag{S18}$$

$$= V^D_{\alpha\beta\gamma\delta}(k_x^\alpha, k_x^\beta)P_{\beta\gamma}(k_x^\beta)c_\alpha^\dagger(k_x^\alpha)c_\delta(k_x^\alpha) - V^E_{\alpha\beta\gamma\delta}(k_x^\alpha, k_x^\beta)P_{\beta\delta}(k_x^\beta)c_\alpha^\dagger(k_x^\alpha)c_\gamma(k_x^\alpha), \tag{S19}$$

where we used the momentum-conserving Kronecker delta and we defined the direct and exchange matrix elements

$$V^D_{\alpha\beta\gamma\delta}(k_x^\alpha, k_x^\beta) = \int_{q_y}\lambda_{\alpha\delta}(k_x^\alpha, k_x^\alpha, q_y)\lambda^*_{\gamma\beta}(k_x^\beta, k_x^\beta, q_y)V(0, q_y), \tag{S20}$$

$$V^E_{\alpha\beta\gamma\delta}(k_x^\alpha, k_x^\beta) = \int_{q_y}\lambda_{\alpha\delta}(k_x^\alpha, k_x^\beta, q_y)\lambda^*_{\gamma\beta}(k_x^\alpha, k_x^\beta, q_y)V(k_x^\alpha - k_x^\beta, q_y). \tag{S21}$$

To this interaction Hamiltonian we add a kinetic component

$$H_{\text{kin}} = \epsilon_\alpha(k_x)c_\alpha^\dagger(k_x)c_\alpha(k_x) \tag{S22}$$

with the phenomenological bandstructure (chosen to reproduce the key features of the bandstructure found in [1])

$$\epsilon_{\tau\eta}(k_x) = W\eta\left(\tau\cos\frac{\pi k_x}{2\kappa} + \frac{1}{5}\sin\frac{\pi k_x}{\kappa}\right) \tag{S23}$$

with W the bandwidth. We now perform self-consistent Hartree-Fock calculations on the Hamiltonian

$$H_{\text{HF}} = H_{\text{kin}} + H_{\text{int,HF}}. \tag{S24}$$

REFERENCES

[S1] P. Sessi, D. D. Sante, A. Szczerbakow, F. Glott, S. Wilfert, H. Schmidt, T. Bathon, P. Dziawa, M. Greiter, T. Neupert, G. Sangiovanni, T. Story, R. Thomale, and M. Bode, Robust spin-polarized midgap states at step edges of topological crystalline insulators, Science **354**, 1269 (2016).
